# Role of artificial intelligence in oncologic emergencies: a narrative review

**DOI:** 10.37349/etat.2023.00138

**Published:** 2023-04-28

**Authors:** Salvatore Claudio Fanni, Giuseppe Greco, Sara Rossi, Gayane Aghakhanyan, Salvatore Masala, Mariano Scaglione, Michele Tonerini, Emanuele Neri

**Affiliations:** 1 Department of Translational Research, Academic Radiology, University of Pisa, 56126 Pisa, Italy; 2 Department of Medicine, Surgery and Pharmacy, University of Sassari, 07100 Sassari, Italy; 3 Department of Surgical, Medical, Molecular and Critical Area Pathology, University of Pisa, 56126 Pisa, Italy; University of Campania “L. Vanvitelli”, Italy

**Keywords:** Artificial intelligence, machine learning, deep learning, neural networks, oncologic emergencies, oncologic imaging

## Abstract

Oncologic emergencies are a wide spectrum of oncologic conditions caused directly by malignancies or their treatment. Oncologic emergencies may be classified according to the underlying physiopathology in metabolic, hematologic, and structural conditions. In the latter, radiologists have a pivotal role, through an accurate diagnosis useful to provide optimal patient care. Structural conditions may involve the central nervous system, thorax, or abdomen, and emergency radiologists have to know the characteristics imaging findings of each one of them. The number of oncologic emergencies is growing due to the increased incidence of malignancies in the general population and also to the improved survival of these patients thanks to the advances in cancer treatment. Artificial intelligence (AI) could be a solution to assist emergency radiologists with this rapidly increasing workload. To our knowledge, AI applications in the setting of the oncologic emergency are mostly underexplored, probably due to the relatively low number of oncologic emergencies and the difficulty in training algorithms. However, cancer emergencies are defined by the cause and not by a specific pattern of radiological symptoms and signs. Therefore, it can be expected that AI algorithms developed for the detection of these emergencies in the non-oncological field can be transferred to the clinical setting of oncologic emergency. In this review, a craniocaudal approach was followed and central nervous system, thoracic, and abdominal oncologic emergencies have been addressed regarding the AI applications reported in literature. Among the central nervous system emergencies, AI applications have been reported for brain herniation and spinal cord compression. In the thoracic district the addressed emergencies were pulmonary embolism, cardiac tamponade and pneumothorax. Pneumothorax was the most frequently described application for AI, to improve sensibility and to reduce the time-to-diagnosis. Finally, regarding abdominal emergencies, AI applications for abdominal hemorrhage, intestinal obstruction, intestinal perforation, and intestinal intussusception have been described.

## Introduction

Oncologic emergencies represent a wide spectrum of conditions comprehending any acute morbid or life-threatening events in oncologic patients either because of the malignancy or because of their treatment and may arise at any time in the natural course of the disease [1, 2]. To correctly recognize oncologic emergencies is essential for prompt and efficient management and to avoid more serious consequences.

Mediastinal syndrome, spinal cord compression, endocranial hypertension, metabolic syndromes, and pulmonary thromboembolism are just a few of the several oncologic emergencies occurring in clinical practice.

The wide spectrum of oncologic emergencies may be classified according to the affected organ system, or may be differentiated, according to the underlying physiopathology, in metabolic, hematologic, and structural conditions [3]. Among these three broad categories, the radiologist has a recognized role and offers game-changing assistance in structural conditions, through an accurate diagnosis useful to provide optimal patient care.

Structural conditions may involve the central nervous system, thorax, or abdomen, and emergency radiologists have to know the characteristics pathophysiology and imaging findings of each one of them [4]. In the last few years, the increasing demand for imaging exams in the emergency department has put rising pressure on emergency radiologists, along with the fact that oncologic emergencies stand out among all the possible emergencies for their complexity due to the high frailty and multiple medical comorbidities of the oncologic patient [5]. Indeed, the number of oncologic emergencies is growing not only due to the increased incidence of malignancies in the general population, but also to the improved survival of oncologic patients because of the advances in cancer treatment [6]. The rapid increase in oncologic emergency incidence and the complexity of these patients account for a thought challenge for the radiology emergency department. Hope lies in the advent of new technologies, such as artificial intelligence (AI), enabling adeptness to assist radiologists with rapidly increasing workloads [7]. AI is a subfield of computer science dealing with the development of tools able to simulate human intelligence, including learning, reasoning, and self-correction [8]. Unfortunately, to our knowledge, the application of AI in the setting of the oncologic emergency is mostly underexplored, probably due to the inherent heterogeneity of the background condition and variability of the acquired data. However, oncologic emergencies are defined by the cause and not by a specific set of symptoms and radiological findings; therefore, there is no gross radiological difference between a pulmonary embolism (PE) caused by a predisposing neoplasm, and one caused by a coagulation disorder. Thus, it can be expected that AI applications developed for PE can be broadly transferred in the clinical scenario of PE caused by cancer. Of course, this transfer is not always for granted, and in some settings, it became much more complex.

**Figure 1.**
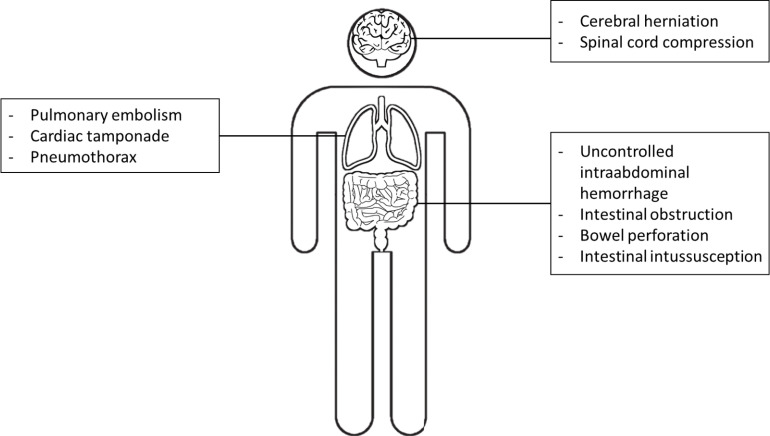
List of central nervous systems, thoracic, and abdominal emergencies currently addressed by AI applications in literature

This narrative review aims to summarize the state-of-the-art AI applications translated to oncologic emergencies, where radiologists play a pivotal role in timely diagnosis that largely impacts patient care. A craniocaudal approach was used and central nervous system, thoracic, and abdominal oncologic emergencies have been addressed regarding the potential applications of AI (Figure 1 and Table 1).

**Table 1.** Characteristics of the oncologic emergencies addressed by AI applications in literature

**Disease**	**Cause**	**Symptoms**	**First-line imaging**	**Imaging features**
Cerebral herniation	Increased intracranial pressure	Headache, vomiting, different level of state of confusion	CT	Midline shift in confront of opposite site, masses
Spinal cord compression	Metastases could compress spinal cord directly or indirectly dislocating vertebral bodies	Back pain, paraesthesia, erectile dysfunction, weakness	MRI	Dislocation of the spinal cord centreline
PE	Tumor invasion of artery branches or increased coagulation effectiveness by therapies or devices	Chest pain, dyspnea, orthopnoea, cough, haemoptysis	CT	Hypodensity or filling defects in the branches of the pulmonary arterial system after contrast
Cardiac Tamponade	Tumors infiltration of pericardium or due to therapies, lymphadenopathy, or infections	Chest pain, dyspnea	CXR/CT	Cardiomegaly and epicardial fat pad sign and in CT high density of pericardial effusion in basal condition (30–45 HU in acute bleeding)
Pneumothorax	Drainage mispositioned or for lung biopsy or tumors infiltration of pleura	Chest pain, dyspnea	CXR	Radiolucent area between the lung parenchyma and the chest wall
Abdominal hemorrhage	Hypervascular neoplasm, splenic rupture due to lymphoma and tumors’ direct vascular invasion	Abdominal pain, asthenia	CT	Increased density of abdominal effusion in basal condition that tends to grow after contrast
Intestinal obstruction	Tumor growth within the intestinal wall or its lumen	Abdominal cramping pain, vomiting, inability to defecate	CXR	Dilatated bowel loops with air-fluid levels
Bowel perforation	Tumor infiltration of intestinal wall	Abdominal pain, rigid abdomen on examination	CXR	Subphrenic free air in frontal CRX and Rigler sign (or the double-wall sign because gas outlines both sides of the bowel wall)
Intestinal intussusception	Invasion of intestinal wall by malignancies	Abdominal pain, nausea or vomiting	CT	Bowel in bowel sign

CT: computerized tomography; MRI: magnetic resonance imaging; CXR: chest X-ray

## AI applications in oncologic central nervous system emergencies

The most common oncologic central nervous system emergencies are cerebral herniation, carcinomatous meningitis, and spinal cord compression [5].

If not promptly recognized and treated, a cerebral herniation may lead to rapid deterioration of the patient’s condition, coma, and death. Cerebral herniation in cancer patients is mostly caused by primary or secondary lesions and massive intracranial hemorrhage. The cerebral herniation through the skull is due to the increase in intracranial pressure [9, 10]. The increase of intracranial pressure can be measured through the midline shifts towards the contralateral site, which can be automatically computed through deep learning (DL)-based algorithms to predict the severity and support the clinical decision. Nag et al. [11] used a convolutional neural network (CNN) to predict the deformed left and right hemispheres on non-contrast CT in patients with epidural and intracranial hemorrhage. As expected, according to the Monro-Kellie hypothesis, the midline shift is an entity well correlated with hematoma volume and a similar result could be hypothesized for cerebral herniation caused by cancer [12]. Besides AI classification algorithms able to detect patients with midline shifts, the prediction models allowing to identify patients at risk of developing cerebral herniation according to either imaging or clinical features, seem to be of greater interest.

In a recent paper, Zeng et al. [13] used an ensemble machine learning (ML) model to predict the risk of cerebral herniation in patients with acute ischemic stroke and obtained an area under the curve (AUC) of 0.904. To optimize the algorithm performance, the authors performed a multivariable analysis combining imaging data from non-contract CT, such as the volume of the hypo-hyperdense lesion, and clinical data like sex, age, smoking, National Institutes of Health Stroke Scale (NIHSS) score, Glasgow Coma Scale (GCS) score, and blood pressure [13]. A similar study could be performed in the oncologic setting, considering imaging variables such as the amount of edema caused by the neoplasm. Spinal cord compression recognizes an oncologic etiology in 5–10% of cases, mostly due to breast, lung, and prostate carcinomas metastasis [6]. The secondary lesion may compress the spinal cord directly encroaching into the spinal canal or indirectly causing the collapse and posterior displacement of vertebral bodies. Similar to midline shift measures for increased intracranial pressure, spinal cord compression may be derived from the mark of the spinal cord centerline after segmentation of the spine regions [14].

In a systematic review of AI methods for imaging of spinal metastasis, Ong et al. [15] described several workflow steps which could benefit from AI implementation, ranging from image acquisition to reporting and integrated diagnostics. Particularly, AI was demonstrated able to precisely provide prognostic information regarding the risk for spinal cord compression due to vertebral fracture after radiotherapy of spinal metastasis or metastatic epidural disease [16].

These tools may be extremely helpful to early identify patients who require prompt surgical or palliative treatments.

## AI applications in oncologic thoracic emergencies

Oncologic thoracic emergencies are broadly categorized into 3 groups: cardiovascular, respiratory, and mediastinal emergencies.

The most relevant cardiovascular emergencies in oncologic patients are PE, cardiac tamponade, and superior vena cava syndrome [5]. PE represents a common disease with a high risk of morbidity and mortality due to an obstruction of one or more pulmonary arteries’ branches of the lung. In oncologic patients, PE may occur due to locoregional tumor seeping in the artery or because chemotherapies induce cancer to release factors that increase coagulation effectiveness. In addition, some devices, like a central vascular catheter, may induce local inflammation reducing blood rate or changing/inducing damage to endothelium [17].

Computed tomographic pulmonary angiography (CTPA) represents the gold standard to diagnose PE but still remains a challenging diagnosis requiring a lot of time and radiologists’ expertise, leading to an increase in error ratio and misdiagnosis [18]. AI could help to detect PE increasing sensibility and reducing the time of diagnosis, leading to early management of this condition and improvement of patients’ outcomes.

First, AI may help the physician to identify patients at high risk of PE requiring medical imaging examination. Indeed, clinical suspicion of PE cannot rely on pathognomonic symptoms. For example, patients could complain of chest pain and dyspnea, and the D-dimer value could be high [19]. Many pathological conditions are characterized by these symptoms and signs like heart attack, pericarditis, pulmonary edema, pneumonia, pneumothorax, and heart failure.

ML has been investigated as a tool able to identify the hospitalized patient at high risk of PE by combining clinical, therapeutic, and laboratory data. It has been shown that the gradient boosted decision trees outperform logistic regression and neural networks, achieving an area under the receiver operating characteristic (AUROC) of 0.85 with high specificity, positive and negative likelihood ratios, and diagnostic odds ratio [20].

The importance of integrating clinical and laboratory data was highlighted by Rucco et al. [21], who implemented a neural hypernetwork analyzing 28 diagnostic clinical features to recognize patients developing PE. The model was integrated into the emergency setting as a first screening tool and achieved a 94% accuracy in predicting PE [21].

In the clinical setting of oncologic patients, many other clinical data could be integrated into the model, such as tumor histotype and site, therapy, vascular devices’ presence, laboratory data, and vital sign measurement. However, to bring AI models closer to clinical implementation, some limitations must be overcome, and prospective multicentric studies are needed to test their real functioning and utility [22]. AI could also assist radiologists in several ways. Many studies already implemented DL algorithms to enhance the radiologists’ workflow by automated triaging and flagging PE on CTPA, which helps to prioritize important cases and hasten the diagnoses for at-risk patients [23–25].

Indeed, early diagnosis and communication to the referring physician may lead to earlier treatment, and to achieve these results and to improve the communication between the radiologist and the clinician electronic notification systems were developed. However, Schmuelling et al. [26] demonstrated that the DL algorithm combined with an electronic notification system did not significantly affect the report reading time or the time to administer treatments.

Alternatively, AI could be used to provide prognostic biomarkers, such as right ventricular to left ventricular (RV/LV) diameter ratio. ML algorithms were developed to automatically compute this parameter from CTPA.

Cardiac tamponade is a severe form of pericardial effusion and is characterized by a profuse accumulation of fluid, usually blood, in the pericardium [27]. In oncologic patients, this pathological condition is due to the tumor’s infiltration of the pericardium, especially in the case of lung, breast, melanoma tumor, or some lymphomas/leukemia [28].

In oncologic patients, cardiac tamponade may occur also due to therapies, infections, or lymphadenopathy. CT is the more accurate imaging modality to diagnose it [29].

In a recent forensic study, DL was used to classify pericardial effusion as hemopericardium or a tamponade caused by other fluids, and to quantify the amount of effusion [30]. The best-performing classification network classified correctly almost all cases of hemopericardium. However, these enthusiastic results have to be confirmed *in vivo* in an emergency setting on electrocardiogram (ECG)-guided CT. Regarding the quantification of the tamponade, the algorithm tended to underestimate the effusion’s quantity [30].

Oncologic patients may suffer from several respiratory emergencies, such as respiratory failure, massive hemoptysis and pneumothorax. Among these, the current evidence supports that pneumothorax gains important benefits from medical image AI analysis. Pneumothorax represents the presence of free air within the pleural space and in oncologic patients may be caused by mispositioning of drainage or other devices like the central venous catheter, or by invasive procedures such as lung biopsy or rarely to tumor infiltrating pleura [31–33].

Pneumothorax requires intervention with needle aspiration or chest intubation, and it can compromise health-related quality of life. The quantification of the amount of pneumothorax is necessary to properly manage the patient. AI is useful to objectively quantify pneumothorax amounts automatically and accurately on CXR and CT scans, leading to a decreased time to diagnosis and treatment, particularly in patients suffering from tension pneumothorax. The amount of pneumothorax is calculated as the radiolucent area between the lung parenchyma and the chest wall on CXR and as the radiolucent volume within the pleural cavity on chest CT [34].

Zhou et al. [35] effectively trained a DL model to identify, segment, and semi-quantify pneumothorax on CXR using the degree of lung compression. Similarly, Kim et al. [36] used the U-net architecture to identify and quantify the pneumothorax on CXR, and the amount calculated by the model did not significantly differs from the volume estimated on CT imaging, defined as the gold standard.

Finally, mediastinal oncologic emergencies include esophageal perforation, acute mediastinitis, and tracheoesophageal fistula. However, to our knowledge, no studies addressed the potential role of AI in these clinical scenarios.

## AI applications in oncologic abdominal emergencies

The most common abdominal oncologic emergencies are uncontrolled intraabdominal hemorrhage, intestinal obstruction, bowel perforation, intestinal ischemia, intussusception, and urinary tract obstruction [5]. Intraabdominal bleeding is a life-threatening complication in oncologic patients. Different causes have been described in the literature, ranging from spontaneous bleeding of hypervascular neoplasm such as renal cell carcinoma, the spontaneous splenic rupture caused by lymphomas or leukemias and large masses with peripheral increased vascularity, to direct vascular invasion [37].

These patients usually present with acute hemoperitoneum, appearing as hyperdense with attenuation values of 45–70 HU on unenhanced CT, and as active contrast medium extravasation at the bleeding site after intravenous contrast administration. However, the identification of hemoperitoneum may be challenging in ultrasound (US) examination, particularly in patients with superimposed ascites.

Lin et al. [38] trained a DL model to identify free fluid on US in a post-traumatic setting. As result, the authors achieved optimal sensitivity, specificity, and accuracy, with slightly lower values for perihepatic and perisplenic effusion, probably due to the lower amount of effusion in these anatomic sites [38]. Hopefully, these results could be transposed to the oncology setting in patients with suspected bleeding, to effectively select patients for contrast-enhanced CT.

Intestinal obstruction in cancer patients usually occurs in advanced gastrointestinal and gynecological malignancies. About 10% to 30% of patients with colorectal cancer and 20% to 50% of those with ovarian cancer develop acute intestinal obstruction. Intestinal obstruction may be caused by a growth within the wall, causing impaired bowel motility and linitis plastica by a growth of the neoplasm within the intestine lumen leading to intraluminal occlusion; or from an extraluminal occlusion such as from serious metastases [39, 40].

In particular, small bowel obstruction is a surgical emergency that can lead to bowel necrosis, perforation, and death. Abdominal X-rays are the first-line imaging test for small bowel obstruction typically demonstrating dilated bowel loops with air-fluid levels. Abdominal multiphasic CT is more sensible, and it is pivotal to confirm the site, severity, and cause of the obstruction and eventually to stage the patient and select the treatment plan [41, 42].

The detection of small bowel obstruction on a conventional radiograph may be challenging, especially for young inexperienced radiologists, and to facilitate it, much research focused on AI applications for this task [43]. In a study by Cheng et al. [43], DL was evaluated to identify small bowel obstruction on conventional radiography. The authors obtain a sensitivity of 83.8% and a specificity of 68.1%. The low value of specificity was caused by the small number of positive cases presented to the neural network. Thus, the authors retrain the model only with images classified positive for bowel obstruction and the specificity increased to 91.9%. Kim et al. [44] assessed several convolutional neural networks and obtained an almost overlapping sensitivity and specificity.

Another relevant abdominal oncologic emergency is intestinal perforations, which are caused by malignancies in 8–10% of perforations in patients with pneumoperitoneum [45]. Bowel perforation can precipitate peritonitis followed by fulminant sepsis and cardiocirculatory shock. Diagnosis of acute abdominal pain usually begins with abdominal radiography, as recommended by the American College of Radiology. The key finding to detect pneumoperitoneum is the presence of subphrenic free air on frontal CXR [46].

Again, CT is more sensitive and allows the detection of the primary neoplasm, the site of the perforation, and in cases of administration of oral contrast material, extravasation of intestinal contents [41]. Although less sensitive, abdominal radiography has significantly lower radioactivity compared to abdominal CT scans, and, for this reason, it is usually utilized in the follow-up of abdominal obstruction and non-obstructive ileus [47, 48]. However, as above-mentioned, many emergency physicians lack sufficient experience to recognize pneumoperitoneum promptly and for this reason, it is essential to develop an automated method for frontal review of the X-ray images of the chest to warn of the danger of the clinical picture and to have a second look.

Su et al. [49] proposed a DL method for alerting emergency physicians about the presence of subphrenic free air on frontal CXR, and achieved 0.875, 0.825, and 0.889 in sensitivity, specificity, and AUC scores, respectively. This tool may provide a sensitive additional screening to detect pneumoperitoneum. Alternatively, pneumoperitoneum may also be detected through the use of artificial neural network (ANN) on abdominal radiographs, leading to increased effectiveness of clinical practice and patient care [50, 51].

Another relevant cause of obstruction is intestinal intussusception, which can be caused by both primary and secondary malignancies involving the wall of the small intestine and colon. Intussusception in adults is rare, and accounts for only 5% of bowel obstructions, but almost 50% of these intussusceptions recognize a neoplastic etiology; among these the most frequent are intestinal lymphoma, gastrointestinal neoplasms, and metastases of the intestinal wall [51]. The CT findings of telescoping or “bowel-in-bowel”, with or without incorporation of mesenteric fat and vessels, are pathognomonic for intussusception [47].

Abdominal radiography has a low sensitivity of about 45% in detecting intestinal intussusception, while US offers both high sensitivity (97.9%) and specificity (97.8%) for detecting intussusception.

Many studies have been carried out to investigate the potential role of AI to increase sensitivity [52] in the diagnosis of intestinal intussusception. DL-based algorithms have been developed for the detection of ileocolic intussusception on abdominal radiography and abdominal US [53–55]. Kim et al. [53] demonstrated that a DL may increase radiologists’ diagnostic performance in detecting intestinal intussusception. Indeed, it is not surprising that the sensitivity of the algorithm was demonstrated higher than that of the radiologists, but it is particularly interesting that the specificity was almost superimposable (0.92 *vs.* 0.96) [54]. Another study by Li et al. [55] assessed the performance of DL for the automatic detection of “concentric circle” signs in US images.

Due to epidemiological issues, all these studies have been conducted on a pediatric population and many challenges have to be addressed to transpose these results to an adult oncologic population. However, many studies have already reported an acceptable performance of AI-based software approved for adults when used on pediatric population, and vice versa the same result could be expected when implementing software designed for pediatric population for adult studies [56].

## Conclusions

In conclusion, this review demonstrated the lack of studies focusing on AI applications specifically aimed at oncological emergency setting. However, many scientific papers concerning the application of AI on superimposable central nervous system, and thoracic and abdominal emergencies have been published. It can be hypothesized that these applications may be transposed in the oncological emergency scenario. Unfortunately, it is not possible to draw firm conclusions regarding the real effectiveness of this transposition, as further studies are needed to investigate the required adaptations. After this premise, it can be expected that AI will be widely tailored and used in the near future to early recognize time-critical oncologic emergencies and to improve patient management.

## Abbreviations

AI: artificial intelligence
CT: computerized tomography
CTPA: computed tomographic pulmonary angiography
CXR: chest X-ray
DL: deep learning
ML: machine learning
PE: pulmonary embolism
US: ultrasound

## Declarations

### Copyright

© The Author(s) 2023.
